# Space-Resolved OH Vibrational Spectra of the Hydration Shell around CO_2_

**DOI:** 10.1021/acs.jpcb.1c06123

**Published:** 2021-12-20

**Authors:** Pavlin D. Mitev, W. J. Briels, Kersti Hermansson

**Affiliations:** † Department of Chemistry-Ångström Laboratory, Uppsala University, Box 538, S-751 21, Uppsala, Sweden; ‡ Uppsala Multidisciplinary Center for Advanced Computational Science, Uppsala University, Uppsala, SE-751 05, Sweden; § MESA+ Institute for Nanotechnology, University of Twente, P.O. Box 217, 7500 AE Enschede, The Netherlands; ∥ IBI-4, Forschungszentrum Jülich, D-52425 Jülich, Germany

## Abstract

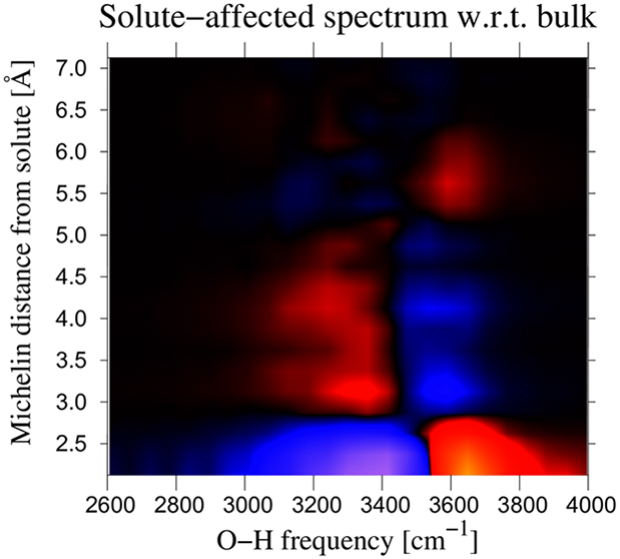

The CO_2_ molecule is weakly bound in water. Here we analyze the influence of a dissolved CO_2_ molecule on the structure and OH vibrational spectra of the surrounding water. From the analysis of *ab initio* molecular dynamics simulations (BLYP-D3) we present static (structure, coordination, H-bonding, tetrahedrality) and dynamical (OH vibrational spectra) properties of the water molecules as a function of distance from the solute. We find a weakly oscillatory variation (“ABBA”) in the ‘solution *minus* bulk water’ spectrum. The origin of these features can largely be traced back to solvent–solute hard-core interactions which lead to variations in density and tetrahedrality when moving from the solute’s vicinity out to the bulk region. The high-frequency peak in the solute-affected spectra is specifically analyzed and found to originate from both water OH groups that fulfill the geometric H-bond criteria, and from those that do not (dangling ones). Effectively, neither is hydrogen-bonded.

## I Introduction

CO_2_–water systems are among the most frequently encountered fluid mixtures in and around the Earth [Duan and Zhang, 2006],^1^ where they govern geological, environmental, and biological processes that affect many aspects of our daily lives. This is reflected in intense R&D efforts taking place in, for example, the area of supercritical CO_2_, the design of “CO_2_-in-water” foams to improve oil extraction processes in the petroleum industry,^2,3^ tentative developments of aqueous Na–CO_2_ and Li–CO_2_ battery systems^4,5^ and in the vast area of carbon capture and sequestration in geological formations and ocean floor sediments.^6,7^ An abundance of experimental and computational studies have been devoted to elucidating the thermodynamic properties, the solubility, and the equation of state of these systems. A summary of many of these efforts has been presented in the Introduction of a recent paper^8^ from the Paesani group.

The present study deals with a very dilute CO_2_−water system, with emphasis on the influence exerted by the solute on the surrounding water. Clearly, also with this particular focus, several papers have already been published. On the theoretical side, we mention statistical simulations with classical force-fields (see, e.g., refs (9−14)), MD simulations with many-body force-fields^8^ generated from quantum-mechanical cluster calculations, as well as simulations that retain the electrons, such as QM/MM-MD simulations^14,15^ or *ab initio* MD (AIMD) simulations.^16−18^ Many of these simulation studies emphasize structural properties, like radial distribution functions, to discuss the featues of the CO_2_ hydration. In addition a number of quantum-mechanical studies of small CO_2_–water clusters, published throughout the years, have added valuable insight concerning CO_2_–water interactions. There seems to be an overall consensus that the carbon dioxide–water interaction is weak, and of a nature that can mostly be classified as hydrophobic.

**Figure 1:**
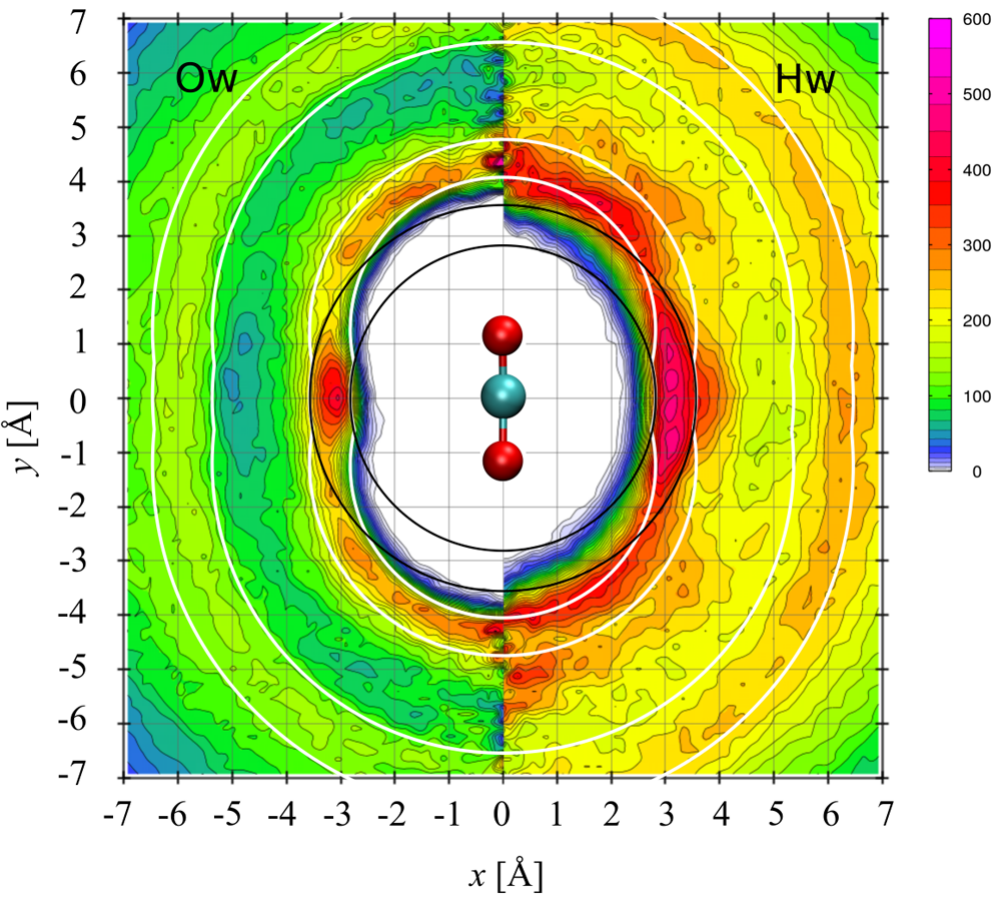
Distribution of water oxygen atoms (left) and hydrogen atoms (right) around a dissolved CO_2_ molecule. Averages are taken over the complete production run of 109 ps. Bins are rectangular tori with cross sections of 0.0233 × 0.0233 Å^2^; contours are according to the corresponding color bars. Since snapshots are taken from a simulation with flexible carbon dioxide (∠OCO) = 180° ± 7°), a local coordinate system was chosen, with the *z*-axis along the bond between carbon and the upper (solute) oxygen. The white lines are chosen to emphasize characteristic shells in the oxygen distribution. Their radii are 2.7, 3.5, 5.3, and 5.5 Å, respectively.

On the experimental side, information about the CO_2_ hydration shell structure from X-ray absorption spectroscopy,^11^ together with thermodynamic information on solubility, point toward the same conclusion. Besides structural and energetic information, also experimental vibrational spectroscopy investigations^18−20^ have been used to conclude that carbon dioxide may be considered to be a more or less inert object when immersed in water. Clearly this conclusion can only be drawn on the basis of indirect inference. It is our aim to shed light on these latter claims by means of an analysis of data obtained with *ab initio* molecular dynamics simulations. The present study therefore naturally emphasizes the influence of the CO_2_ solute on the vibrational properties of water molecules in its neighborhood. We will present some structural results as well, but mainly to have them available when discussing the origin of the observed variations of water vibrations in the hydration shell. A quick impression of density variations near the solute can be obtained from Figure 1. The white lines in this figure are a guide to the eye to delineate characteristic regions. They are the azimuthal projection of surfaces of constant “Michelin” distances (see below).

When discussing vibrational spectra, we will make extensive use of calculated weighted vibrational difference spectra, to display the perturbation caused by the CO_2_ solute on the vibrational signatures of the solvent molecules. The method has been developed and successfully applied in experimental vibrational spectroscopy. It was first introduced as the “double-difference method” by Lindgren et al.^21,22^ to extract the OH spectrum originating from the first hydration shell around cations and anions in aqueous solution. Stangret et al.^23^ developed a related approach and exploited the possibility of gathering further information from measurements on a larger number of concentrations. Separately and partly together, these groups singled out the signatures of the hydration shells of a whole range of cations and anions, and reported their OH peak frequency values, as listed, e.g., in ref (24). For a different class of solutes, Ben-Amotz and co-workers developed an essentially similar strategy based on Raman measurements and multivariate curve resolution (MCR) analysis to single out the “solute-affected” spectrum^25^ from a solution spectrum and characterize the solvation shell around organic molecules in aqueous or organic solvents. In a collaborative experimental–computational effort,^18^ this approach was turned into a *tool* to provide evidence for a phase transition in the water layer surrounding a CO_2_ molecule at ambient temperatures, that would otherwise have been very difficult to obtain. Our analyses in the current paper build on the same AIMD simulation trajectories that were used in ref (18). Those simulations were performed with the BLYP-D3 density functional, i.e., with dispersion interactions taken into account. This is a logical approach as the water–CO_2_ interaction is weak. For more information, see section II.A.

In the vibrational analysis presented in the current paper, we present three complementary types of spatially resolved vibrational spectra. Such spectra are essentially inaccessible by experiments. Thus, for example, *power spectra for open systems* are presented for consecutive “slices” around the solute (the slices adhering to the shape of the solute). We will show that by tiling these spectral slices together and subtracting the standard OH power spectrum of bulk water we obtain a difference spectrum that displays a shell-like variation of the OH frequency as a function of distance from the CO_2_ solute. Moreover, we observe an accompanying weak but significant variation of the water–water H-bond structure.

## II Methods

### II.A *Ab Initio* Simulations

We carried out *ab initio* molecular dynamics (AIMD) simulations of carbon dioxide dissolved in liquid water. As a reference system, we also ran a simulation of pure water. The trajectories from these simulations were used in ref (18), but here we present additional analyses and conclusions based on these data. We repeat the main details of the simulations here.

Pure water was modeled by 111 H_2_O molecules in a cubic box of length 14.917 Å with periodic boundary conditions, corresponding to a density of 1.00 g per cubic centimeter. The aqueous solution was created by removing one water molecule and replacing it by a CO_2_ molecule giving a 0.5 m solution.

The electronic structure calculations were carried out using the hybrid Gaussian plane-wave method^26^ as implemented in the QUICKSTEP/CP2K code.^27^ We used triple-ζ valence doubly polarized (TZV2P) basis sets, which have previously been shown to provide a good compromise between accuracy and computational cost. The BLYP exchange correlation functional was used and supplemented with the Grimme-D3(0) dispersion correction,^28^ and the core electrons were treated by the Goedecker–Teter–Hutter norm-conserving pseudopotentials.^29,30^ Our molecular dynamics simulations were of the Born–Oppenheimer type and were carried out in the *NVT* ensemble with a Nosé–Hoover thermostat chain, targeted at a temperature of 323 K, and a time step of 0.25 fs. We chose this functional and computational setup because they have been thoroughly investigated by Jonchière et al.^31^ in their work on water, with generally good results. Those authors found, however, that the theoretical phase diagram of water was shifted in temperature by about 25 deg with respect to the experimental phase diagram and that water in its liquid state at room temperature was well described by simulations at 323 K and a density of 1.00 g/cm^3^. In our simulations, averages were collected from runs of 127 ps for pure water and 109 ps for the CO_2_(aq) system, preceded by 10 ps equilibration in both cases in order to achieve reasonable dynamical equilibrium.

**Figure 2:**
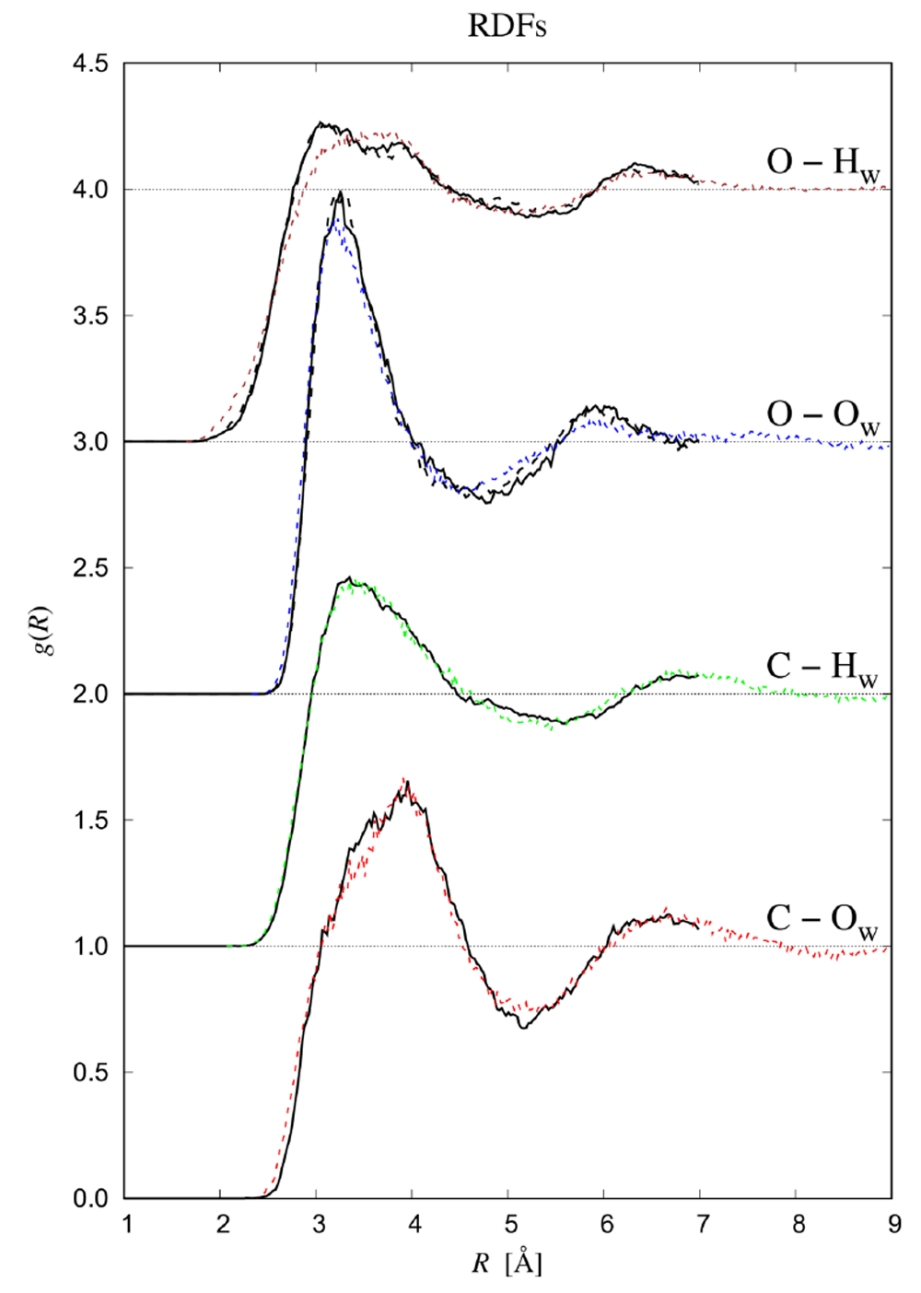
Atom–atom pair distribution functions resulting from our BLYP-D3 AIMD simulation (black solid lines), compared with those from the MD simulation by Riera et al.^8^ performed with the many-body potential energy function MB-nrg based on high-level quantum-mechanical calculations (dashed colored lines).

As an additional assessment of the quality of our computational methods we have calculated various atom–atom radial distribution functions (rdf) and plotted them in Figure 2 together with corresponding rdfs from Riera et al.^8^ The latter were obtained with MD simulations using many-body potentials fitted to CCSD(T) calculations on a large class of mixed and pure molecular clusters containing up to four molecules of CO_2_ or water. Given the high quality of the quantum mechanical calculations, the respectable sizes of the training sets and the molecular clusters, and the in-depth analysis of the many-body fit-functions, these potentials must be considered to form a benchmark for future models. It is therefore satisfying, and slightly surprising given the substantial differences in quantum mechanical methods used in the two studies, to note that the agreement is excellent. The small difference in densities between the two simulations has no influences on distances. Comparisons with other MD studies of dilute CO_2_(aq) solutions do not display the same excellent agreement with our rdfs as we found in Figure 2, although an overall agreement is generally achieved. This holds for the DFT-based MD simulations of Leung et al.^16^ and Kumar et al.^17^ and also for the Amber force-field based simulations of England et al.^10^ with some rdf curves reported in Lam et al.^11^ Only the Hartree–Fock-based QM/MM-MD simulation results of Moin et al.^15^ differ qualitatively from our results.

### II.B Analysis

Common structural and dynamical properties such as radial distribution functions (rdf) and velocity autocorrelation functions (vac(t)) were calculated using the TRAVIS program package.^32^ Power spectra were obtained by normalizing the vacs such that their values at time zero were equal to one, and next Fourier transforming them.^33^ Notice that vac(t) is a single particle quantity. Modifications made to calculate vacs for open subvolumes of the full simulation box are described later in this section.

#### Michelin Regions

In order to facilitate a more intuitive description of the hydration structure, we use the concept of Michelin distances and regions defined by them. Thus, the Michelin distance *r*_*M*_ between the solute molecule and an arbitrary point is the shortest of the distances from the point to the solute’s three nuclei. The set of points with Michelin distances equal to *r*_*M*_ defines a Michelin surface, and points with Michelin distances smaller than *r*_*M*_ define a Michelin region with volume *V*(*r*_*M*_). A few such shells are marked in Figure 1, where they are delineated by white lines. A *Michelin shell* is the region between two Michelin surfaces with different radii, and it has volume *ΔV*(*r*_*M*_,*Δr*_*M*_) = *V*(*r*_*M*_ + *Δr*_*M*_) – *V*(*r*_*M*_).

#### Velocity Autocorrelation Functions and Power Spectra for Open Systems

As far as dynamics is concerned, we will concentrate on the velocity autocorrelation functions of the hydrogen atoms in our systems. All power spectra mentioned in the Results are obtained through Fourier transformation of these functions. Since translational and orientational diffusion of water molecules involve much longer time-scales than their normal mode vibrations, the characteristics of the latter in frequency space are well separated from the former. As a result, our spectra at high frequencies may be compared with experimental IR and Raman spectra.

In this paper, we will make use of velocity autocorrelation functions for open systems, here for part of the complete box. Since hydrogen atoms may enter and leave the volume under investigation, the individual time series used to calculate the velocity time autocorrelation functions are of variable lengths. The correlator that we use to calculate vac(*t*) chooses with every trajectory *i* of length *T*_*i*_ a maximum correlation time τ_*i*_ ≤ *T*_*i*_. The corresponding velocity time autocorrelation function vac_*i*_(*t*) is then calculated according to
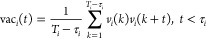
1where *v*_*i*_(*k*) is the velocity of trajectory *i* at time *k*. Notice that we use for all *t* < τ_*i*_ the same number of samples. The average vac(*t*) is then calculated as the average of these vac_*i*_(*t*) with weight proportional to *T*_*i*_ – τ_*i*_:
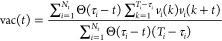
2Here *N*_*t*_ is the total number of trajectories used in the analysis. The Heaviside function Θ(τ_*i*_ – *t*) ensures that only trajectories are taken for which τ_*i*_ > *t*.

Once vac(*t*) is obtained, it is normalized such that its value at *t* = 0 is equal to one, after which the result is Fourier transformed, yielding the power spectrum *D*_*solution*_(ω; *r*_*M*_).

#### Weighted Difference Spectra

As has been done before to analyze experimental spectra,^18,21−25^ we quantify the influence of the solute on the dynamics of the hydrogen oscillators of water in the neighborhood of the solute by partitioning the calculated spectra into two contributions, one from bulk-like water molecules and one from so-called solute-affected water molecules. In order to take full advantage of the detailed information obtained with particle based simulations, we apply the definition to Michelin regions of various sizes indicated by *r*_*M*_. We define the number of solute-affected hydrogen oscillators, *N*_*aff*_(*r*_*M*_), in that volume, and the corresponding solute affected power spectrum, *D*_*aff*_(ω; *r*_*M*_), according to
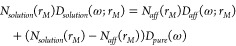
3Here *D*_*solution*_(ω; *r*_*M*_) is the average spectrum obtained with all *N*_*solution*_(*r*_*M*_) hydrogen atoms in the Michelin region under consideration; for its normalization see the previous item in this section. *D*_*pure*_(ω) is the average spectrum of pure water. Since *N*_*solution*_(*r*_*M*_) and *D*_*solution*_(ω; *r*_*M*_) are obtained from the simulation, and *D*_*pure*_(ω) is a known function (obtained from a simulation of bulk water), the above equation contains two unknowns, *N*_*aff*_(*r*_*M*_) and *D*_*aff*_(ω; *r*_*M*_). This means that with any given value for *N*_*aff*_(*r*_*M*_), the corresponding spectrum *D*_*aff*_(ω; *r*_*M*_) can be obtained from the data. In general the affected spectra obtained in this way will not be strictly positive, which is physically nonsensical. The procedure then is to let the smallest value of *N*_*aff*_(*r*_*M*_) for which *D*_*aff*_(ω; *r*_*M*_) is nowhere negative be the physically relevant number of affected molecules in the region out to *r*_*M*_. The corresponding *D*_*aff*_(ω; *r*_*M*_) will be called the solute-affected, or simply, the affected spectrum of the Michelin region around the solute out to Michelin radius *r*_*M*_. Similarly, *N*_*aff*_(*r*_*M*_) will be called the number of affected hydrogen oscillators in that region. The procedure just described is the same as has been used in experimental studies,^18,21−25^ where eq 3 was applied to the full illuminated region. Similarly, we too will sometimes apply the equation to the full simulation box. The challenge for experimentalists is to choose solute concentrations which are small enough for solute-molecules not to influence each other, and at the same time large enough to yield a good signal-to-noise ratio.

A quick comment on the meaning of this analysis is in order. As is clear from eq 3, we consider only two types of spectra, the spectrum of a “pure water molecule” and the spectrum of an “affected water molecule”. As emphasized above, *D*_*pure*_(ω) is the average spectrum of a collection of oscillators characteristic of pure water. Similarly, *D*_*solution*_(ω; *r*_*M*_) is the average spectrum of a collection of oscillators characteristic of the solution in the region under consideration, a collection that is different from that of pure water. *D*_*aff*_(ω; *r*_*M*_) is then a single property to quantify how much these collections differ when it comes to their spectra.

As a first, rather rough, function to picture the influence of the solute on the dynamics of the hydrogen oscillators at position *r*_*M*_, we introduce the fraction, *P*_*aff*_(*r*_*M*_), of affected oscillators in a thin shell at Michelin distance *r*_*M*_ from the solute, according to
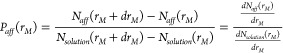
4*P*_*aff*_(*r*_*M*_) may be interpreted as the probability that a hydrogen oscillator in a sufficiently thin Michelin shell at a distance *r*_*M*_ belongs to the class of molecules that give rise to *D*_*aff*_(ω; *r*_*M*_). Like with radial distribution functions, this definition is most informative in case the dynamics of the hydrogen oscillators is reasonably uniform over Michelin shells. For most solute molecules, including CO_2_, this is of course not strictly the case, but the Michelin shells certainly improve the situation compared to the usual spherical shells.

## III Results

We will now discuss the influence of the solute on the OH-stretch dynamics of the water molecules in its neighborhood. The OH-stretch vibration is a very sensitive probe of the chemical environment of the corresponding water molecule. Apart from very close to the solute we do not expect large *direct* influences of the solute but mostly *indirect* influences through changes of densities and hydrogen-bonding. So, indirectly hard-core interactions, being the cause of density oscillations around the solute, will be important in determining the dynamics of the H atoms.

### Structural Properties

Figure 1 displays the density of water oxygen atoms around a CO_2_ molecule in the left half of the figure, and that of the corresponding hydrogen atoms in the right half. Details of the calculation are given in the caption. Since we are dealing with a flexible solute, the *z*-axis of the plot has been chosen along the bond from carbon to the upper solute oxygen atom. The very small differences between the upper and lower halves of the figure give an indication of the quality of the statistics. The white lines in the figure are drawn at Michelin distances chosen to visually display shells with roughly uniform oxygen densities; i.e., the lines were placed to accentuate the gross features of the oxygen distribution in the left part of the figure. The way the same lines also capture the gross features of the hydrogen distribution in the right-hand side of the figure is an interesting observation in itself, and it tells us a lot about the structure and orientation of the water molecules. On the oxygen side, we clearly recognize two shells of excess hydration with a depletion zone in between. To a good approximation, oxygen and hydrogen densities are homogeneous within thin Michelin shells. The same holds true for other properties, to be mentioned below. When discussing velocity power spectra of hydrogen motion (see below), it will in general not be possible to obtain a similar resolution as the one in Figure 1, and we must resort to calculating averages over larger regions. On the basis of the homogeneity of local properties within Michelin shells, we consider averaging over Michelin shells to be the best solution.

**Figure 3:**
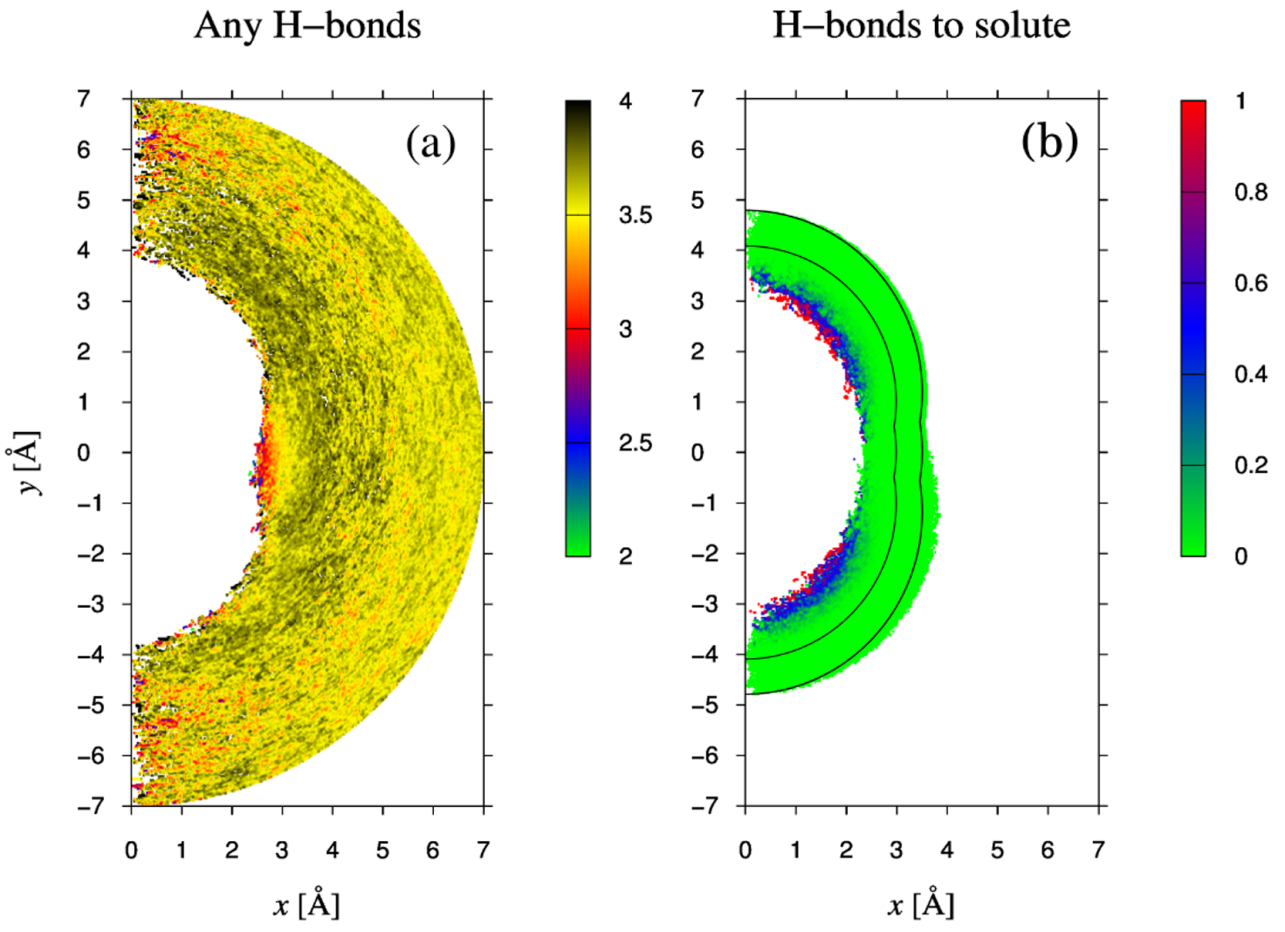
Distribution of hydrogen bonds around the solute. (a) Average number of hydrogen bonds per water molecule, either to another water molecule, or to the solute. (b) Only hydrogen bonds donated to the solute. The color code in panel b is such that green refers to no hydrogen bond and red indicates that all water molecules in this bin are involved in a hydrogen bond to the solute. Data are shown for water molecules within a Michelin radius less than 3.5 Å. In both plots, bins are square tori with cross section of 5.44 × 10^–4^ Å^2^. Positions are taken to be the oxygen positions.

We now consider more closely the distribution of *hydrogen bonds* around the solute. We do so by comparing two types of H-bond coordination numbers, obtained either by counting for each water molecule all hydrogen bonds that it donates to CO_2_ or another water molecule and those accepted from another water molecule (Figure 3a) or by counting only hydrogen bonds donated to the solute (Figure 3b). The hydrogen bond criteria used in this paper are as follows: a particular O–H···O configuration qualifies as a hydrogen bond in the case that both *R*_O···O_ < 3.5 Å and the ∠O···O–H angle < 30°. It should be noted that the pixels displayed in Figures 3 refer to oxygen positions. (This explains why the inner contour near the solute’s waist in Figure 3a is similar to that of the oxygen atoms in Figure 1.) Near the waist, the water molecules closest to the solute are involved in about three hydrogen bonds. None of these are hydrogen bonds to the solute, as may be inferred from the red waters near the waist in Figure 3a being absent in Figure 3b. The few hydrogen bonds that do occur between water and solute are all with water molecules that reside very close to the oxygen atoms of CO_2_ as seen in Figure 3b. Interestingly, the density of water–solute H-bonds are larger in the northeast (and southeast) of the figure than in the north (and south). This is an indication that the hydrogen atoms in the north are located such that the water dipole points toward the solute’s oxygen atom, which makes ∠O–H···O too large to qualify as a hydrogen bond. In the northeast, water molecules may donate a hydrogen atom more directly to the solute’s oxygen, while still having a lot of orientational freedom to interact favorably with the remaining water molecules.

The reader should not be misled by the colorful appearance of the pixels in Figure 3b, since the plot only gives a conditional property; i.e., out of those water molecules which are found in a particular bin the *percentage* that has a hydrogen bond to the solute is displayed. Even when this percentage is equal to 100, there may still be only very few waters that are H-bonded to the solute, as indeed is the case here. To give an impression of the latter, we mention that out of all hydrogen atoms within a Michelin radius of 3.5 Å of the solute, on average only 0.244 hydrogen bonds with the solute occur per time frame. The average lifetime of these hydrogen bonds was calculated to be 0.05 ps, slightly longer than 0.01 ps in ref (17) or 0.02 ps in ref (15). This implies that a hydrogen bond with the solute is formed every 0.2 ps, which then lives for 0.05 ps.

Finally, we consider H-bond coordination numbers at distances larger than 2.7 Å. As seen in Figure 3a, H-bond coordination numbers are slightly above average in the first solvation shell. Contrary to the hydrogen number density, the density of hydrogen bonds simply decays to its bulk value, and does not have the characteristic ripples that result from hard-core interactions.

**Figure 4:**
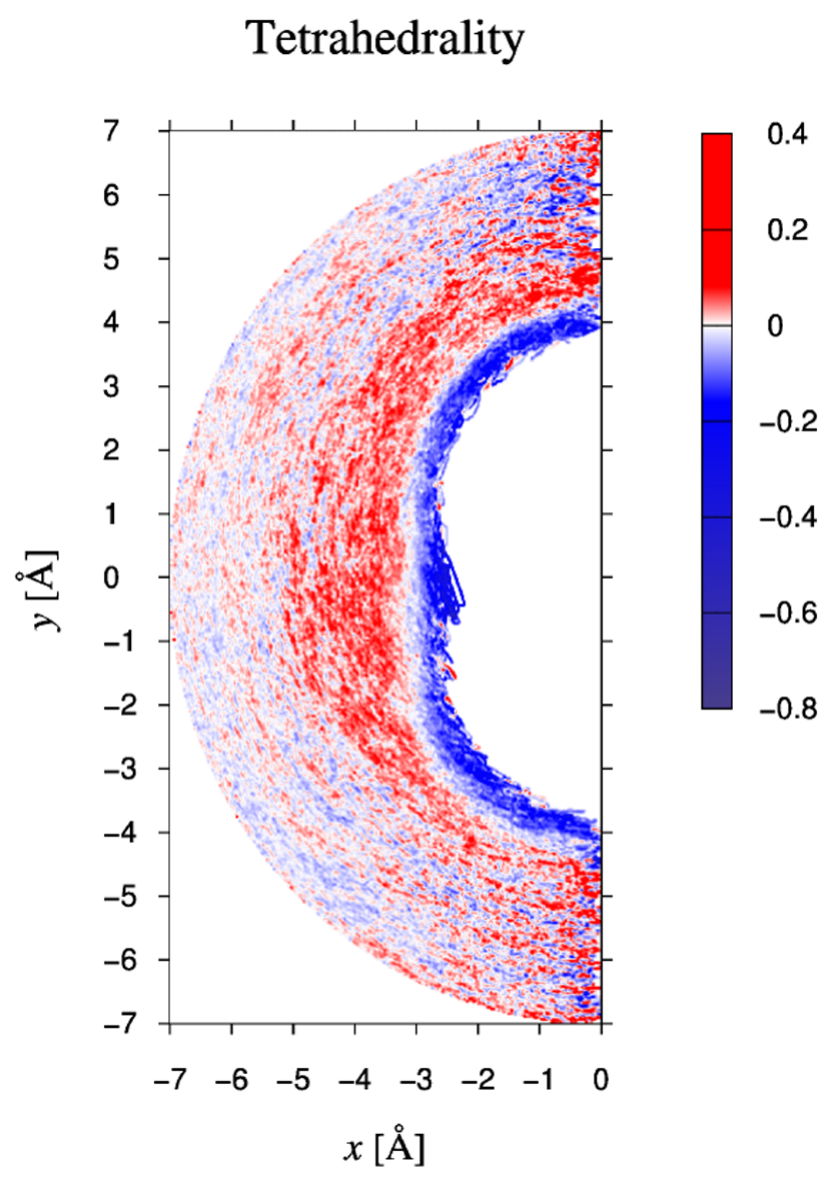
2D map of the tetrahedrality of the local structure around the water molecules. For each bin, the molecular tetrahedrality *Q*_k_ has been averaged over all water molecules *k* in the given bin (cf. eq 5), giving Q. Plotted are values of *Q* – *Q*_*water*_ with *Q*_*water*_ = 0.67. Bins are square tori with intersection 0.0233 × 0.0233 Å^2^ = 5.44 × 10^–4^ Å^2^. Values run from −0.76 to +0.34.

As an additional characteristic of the oxygen structure in the solution around the solute, we have calculated the *tetrahedrality*, *Q*_*k*_.^34,35^ According to its name, tetrahedrality quantifies in a rotationally invariant way, the similarity of the molecule’s immediate neighborhood to a purely tetrahedral environment. Results are plotted in Figure 4 in the form of *Q* – *Q*_*water*_ with *Q*_*water*_ = 0.67 being the average tetrahedrality in our BLYP-D3 bulk water. For details see the caption of Figure 4. *Q*_*k*_ of water oxygen *k* is defined as
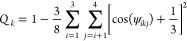
5ψ_*ikj*_ is the angle between water oxygen atoms *i* and *j* as seen from oxygen atom *k*, and the sums *i* < *j* run over the six pairs that can be selected from the four nearest water oxygens within a distance of 4.0 Å from atom *k*. In the case where no four neighbors can be found within the given range, molecule *k* is left out from consideration. In the case where all angles are equal to the tetrahedral angle *Q*_*k*_ = 1, while *Q*_*k*_ = 0 in the case where all cos(ψ_*ikj*_) values are randomly distributed. When applied to MD simulations of ice *I*_*h*_, the average value of *Q*_*k*_ has been found to be 0.99 with a very narrow distribution.^36^

It is clearly seen that tetrahedral order is enhanced at Michelin distances between about 3.5 and 5 Å. This region encompasses the broad density minimum of water oxygen atoms in the left part of Figure 1. Our result is in agreement with the fact that tetrahedrality is prominent in ice which has a density slightly below that of liquid water and with the simulation results of Chiu et al.,^36^ who found that low-density amorphous ice has a *Q*_*k*_ value vastly higher than that of high-density amorphous ice.

### Spectra

**Figure 5:**
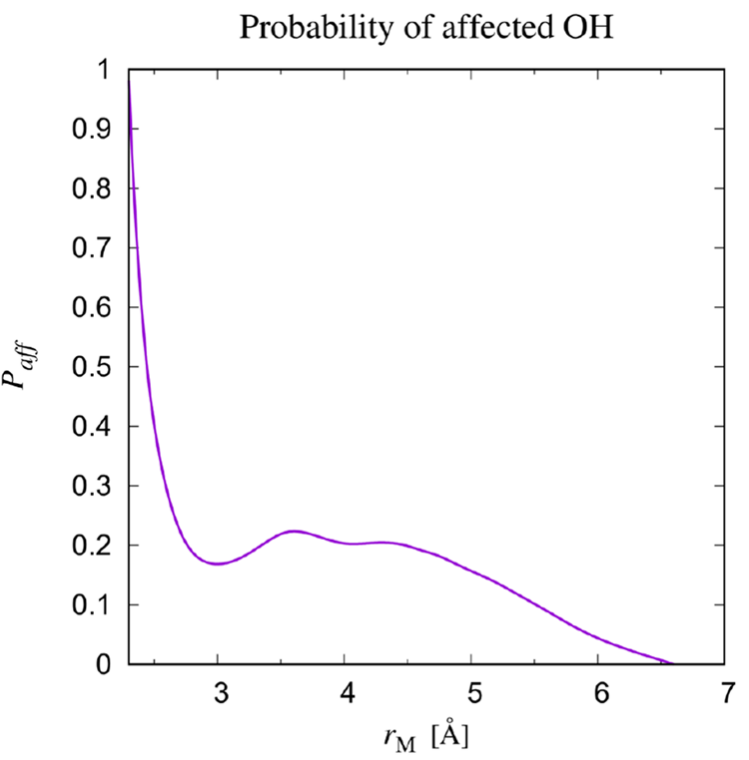
Probability *P*_*aff*_ that a hydrogen oscillator is affected by the presence of the CO_2_ molecule, plotted as a function of the Michelin distance from that molecule. The position of the oscillator is taken to be that of the hydrogen atom. The horizontal axis starts at 2.25 Å and refers to the Michelin distance from the solute.

As a first global measure of the influence of the solute on the vibrational spectra of the water molecules in its neighborhood, we have plotted in Figure 5 the probability *P*_*aff*_(*r*_*M*_) that we defined in eq 4. Recall that *P*_*aff*_(*r*_*M*_) is the fraction of oscillators (in a thin shell near *r*_*M*_) that belong to the class of *affected* oscillators. See the discussion at the end of Section II.B. When calculating power spectra and derived quantities like *P*_*aff*_(*r*_*M*_), we used the instantaneous position of the hydrogen atom to decide whether the oscillator was closer to the solute than the Michelin distance *r*_*M*_ or not.

Two main features may be distinguished. First, the large values of *P*_*aff*_(*r*_*M*_) for very small Michelin radii, such as *r*_*M*_ ≤ 2.7 Å, imply that those oscillators that temporarily reside very close to the solute are almost all affected by the presence of the solute; i.e., their spectrum is different from that of bulk water. From a comparison with the hydrogen-bond count in Figure 3b, we infer that, according to our geometric criterion for hydrogen-bonding, these are the hydrogen atoms with a large probability to hydrogen-bind to one of the oxygen atoms of the solute. The effect quickly decays until *P*_*aff*_(*r*_*M*_) reaches a minimum at a Michelin distance of 3 Å from the solute. Second, beyond this minimum *P*_*aff*_(*r*_*M*_) rises again to reach a plateau value of about 0.2 for Michelin distances between 3.5 and 5.5 Å, after which *P*_*aff*_(*r*_*M*_) gradually decays to zero. Notice that there is a little dip in the plateau near 4 Å. It is striking that the minimum between these two features occurs at distances where the tetrahedrality of the water oxygen atoms (see Figure 4) is about equal to that of pure water.

**Figure 6:**
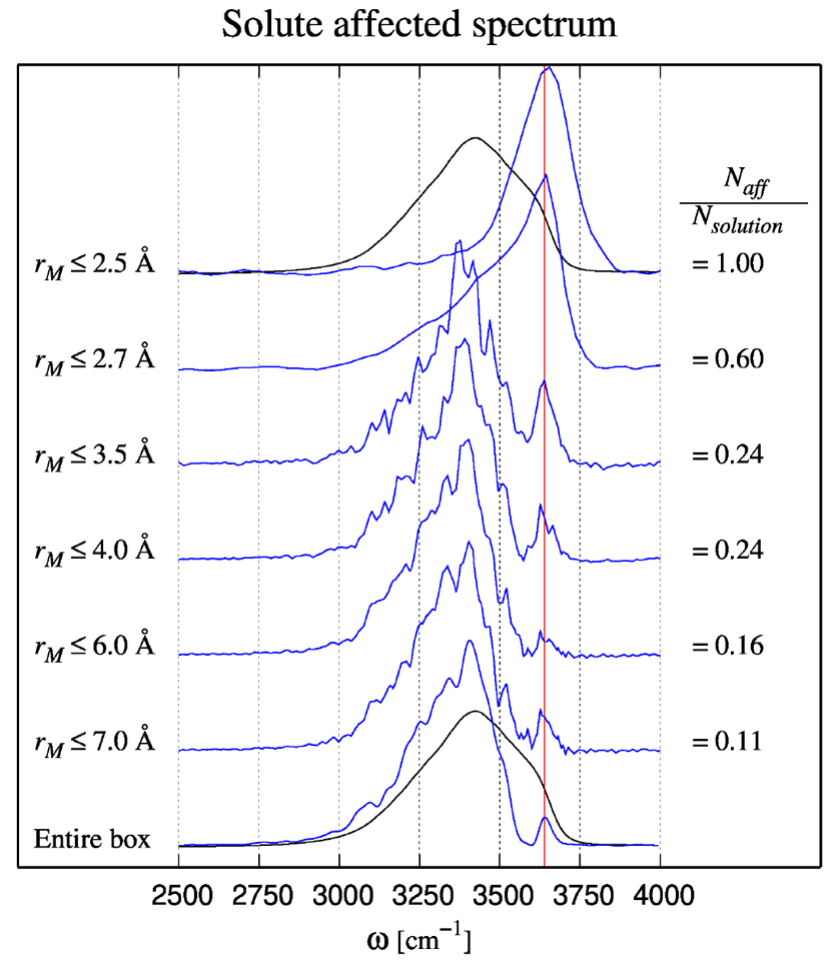
Spectra *D*_*aff*_(ω; *r*_*M*_) for various Michelin volumes with radii indicated. The ratio *N*_*aff*_(*r*_*M*_)/*N*_*solution*_(*r*_*M*_) is listed to the right of each spectrum. The reason that the solute-affected spectrum becomes less noisy with decreasing Michelin radius is that for the smaller volumes, the affected spectrum is basically equal to the full spectrum, reflected by the fact that for *r*_*M*_ = 2.5 Å the *N*_*aff*_(*r*_*M*_) /*N*_*solution*_(*r*_*M*_) ratio is ≈1, while for the entire box, the affected spectrum is only a small contribution to the full spectrum, reflected in the ratio being small. With decreasing Michelin volumes a sharp peak near 3650 cm^–1^ becomes dominant. Black lines represent the spectrum of pure water.

In order to obtain better insight in the type of influence that the solute has on the hydrogen dynamics in the solvation shell, we present in Figure 6*solute-affected* spectra, *D*_*aff*_(ω; *r*_*M*_), for a series of Michelin volumes with different radii. We restrict ourselves in this plot to a frequency range between 2500 and 3800 cm^–1^, which encompasses Raman-measured frequencies characteristic of OH-stretch oscillators.^18^ Data at lower frequencies than these are dominated by contributions from other vibrational modes, such as diffusive translations and rotations of the molecule as a whole. In our protocol for calculating spectra, while varying *N*_*aff*_(*r*_*M*_) in order to have *D*_*aff*_(ω; *r*_*M*_) positive definite everywhere (see Section II.B) of course the whole spectrum at all frequencies from 0 to 3800 cm^–1^ was considered.

From the top to the bottom in Figure 6, solute-affected spectra are shown for Michelin regions of increasing Michelin radius *r*_*M*_. As mentioned at the beginning of this section, the instantaneous hydrogen position determines whether the oscillator belongs to the given region or not. The bottom curve was obtained with oscillators from the entire box. For comparison, the spectrum of pure water is included as a black line, and repeated in the top panel as well. The main difference between the spectrum of pure water and the total spectrum, i.e., the main intensity of the affected spectrum, occurs at frequencies between 3000 and 3600 cm^–1^. Besides this, there is a small peak at about 3650 cm^–1^. With decreasing Michelin volume, these characteristics remain similar until the smallest volumes are considered.

We now concentrate on the top curve in Figure 6, which presents the affected spectrum of a Michelin region with radius *r*_*M*_ = 2.5 Å. Since *P*_*aff*_(*r*_*M*_) in this region was found to be very close to unity, see Figure 5, the top curve in Figure 6 represents at the same time the affected as well as the total spectrum. So, the top spectrum corresponds to a mixture of oscillators hydrogen-bonded (according to our geometric criterion) to an oxygen of CO_2_ (see Figure 3b), plus a few dangling bonds, and possibly some that are hydrogen-bonded to other water molecules. Note that OH-oscillators near the waist are associated with distances larger than 2.5 Å and therefore do not contribute to the spectrum that we are discussing now.

In order to provide additional, *qualitative* understanding of the top spectrum, we assume that all oscillators in this collection move independently from each other. Their spectrum is then just the sum of smoothed delta-peaks, one for each oscillator in the collection, and may be interpreted as the probability density function (pdf) over frequencies of the independent oscillators. We assume that the delta-peak frequencies may to a good approximation be calculated using uncoupled bond-stretching calculations,^37^ meaning that the bond under investigation is stretched and contracted in a frozen environment and the corresponding electronic energy is calculated using the same quantum chemical model as described in Section II.A. Using the molecular energy values along the scans to provide Born−Oppenheimer surfaces on which the oscillators move, harmonic and anharmonic frequencies can be calculated. It has been argued before^38^ that near room temperature, anharmonic corrections are not captured in power spectra of OH vibrations, which should therefore best be compared with harmonic bond-stretching spectra.

**Figure 7:**
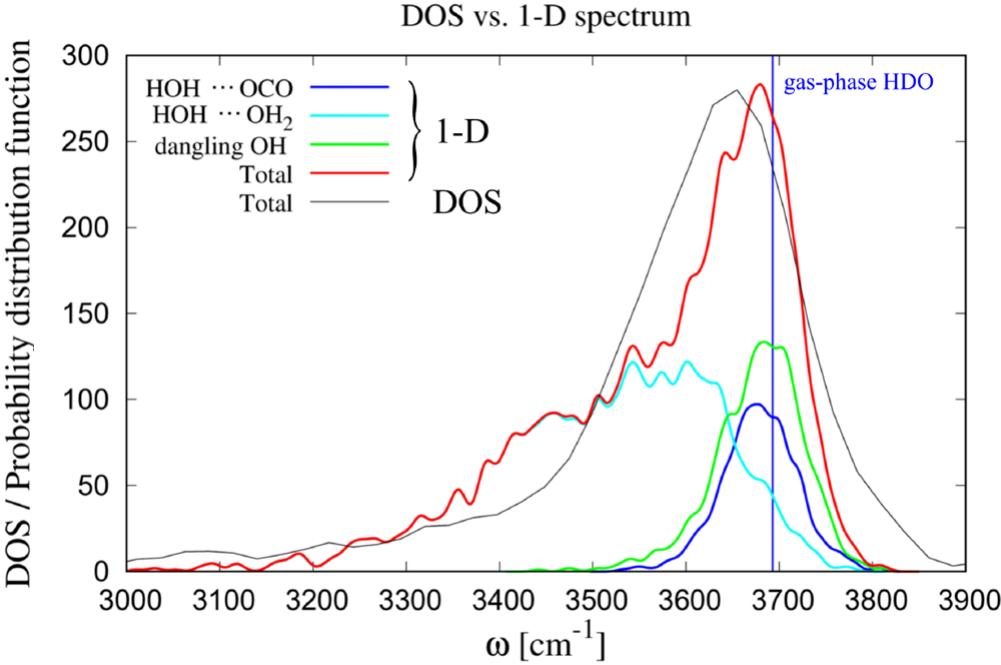
Decomposition of the solute-affected power spectrum *D*_*aff*_(ω; *r*_*M*_ =2.5 Å) into contributions from different OH oscillator categories. Black line: spectrum *D*_*aff*_(ω; *r*_*M*_) = *D*_*total*_(ω; *r*_*M*_) for H-oscillators within a Michelin radius of 2.5 Å from CO_2_. Red line: bond-stretching spectrum obtained with 1581 H atoms out of frames taken every 50 time steps during the whole run. Based on our geometric H-bond criteria, the red spectrum has contributions from 843 (25%) dangling H atoms (green line), 547 (16%) H atoms hydrogen-bonded to CO_2_ (purple line), and 1991 (59%) H atoms hydrogen-bonded to water molecules (light blue line). For reference, a vertical blue line is drawn at the position of the calculated gas-phase harmonic frequency.

We have generated a bond-stretching spectrum by calculating harmonic frequencies for all oscillators in the selected collection (*r*_M_ < 2.5 Å), and plotting the sum of corresponding smoothed delta functions in Figure 7. Clearly the spectrum obtained in this way (red line) is not a perfect representation of the power spectrum (black line), also called density of states (DOS). In spite of such differences, we judge the agreement between the two spectra reasonable enough to accept the bond stretching method as an aid for interpreting the power spectrum. In the figure, we distinguish three contributions to the total spectrum: contributions from H atoms that are, according to our geometric criteria, hydrogen-bonded to the solute (purple line), contributions from H atoms hydrogen-bonded to water molecules (light blue line), and those from dangling H atoms (green line). Both the DOS and the bond-stretching spectrum have their maximum at frequencies that are slightly down-shifted with respect to the gas-phase harmonic value (3693 cm^–1^), with the DOS being down-shifted twice as much as the bond-stretching spectrum. At the frequencies near its maximum, the bond-stretching spectrum is almost completely resulting from dangling H atoms and from those which are hydrogen-bonded to CO_2_. As expected, the dangling bond contribution more or less has a Gaussian shape with its maximum at the gas-phase frequency. The contribution of the H atoms hydrogen-bonded to the solute may be considered to be slightly shifted to lower frequencies, but the statistics does not seem good enough to draw definite conclusions. The similarity between the contributions from these two classes indicates that from an electronic point of view, the alleged hydrogen-bonding to CO_2_ has only very little effect on the calculated spectra. This is in strong contrast to the contribution from those H atoms within 2.5 Å from the solute that are hydrogen-bonded to other water molecules; their harmonic frequencies are severely down-shifted with respect to the gas-phase value as is also the case in bulk water. It seems that our geometric criterion to decide about hydrogen-bonding to the solute does not necessarily imply any electronic differences between different classes.

Returning now to Figure 6, we next slightly enlarge the volume considered for calculating the spectrum to a Michelin radius of 2.7 Å. As was already suggested by the results in Figure 5, the fraction of affected oscillators goes down dramatically. Two interesting effects can be observed in this spectrum. First, the peak around 3650 cm^–1^ narrows substantially, and second, the spectrum has developed a substantial intensity in the frequency range between 3000 and 3600 cm^–1^. Including increasingly more of the solvent by increasing the volume even further, going down in Figure 6, the peak at 3650 cm^–1^ in the affected spectrum continues to narrow and decrease, while at the same time the intensity in the frequency range from 3000 to 3600 cm^–1^ increases until it reaches a stable value for radii beyond about 6 Å.

As mentioned before, the peak around 3650 cm^–1^ continues to narrow with increasing Michelin volume used to calculate the spectra. This is somewhat surprising. In principle the oscillators that were responsible for the spectrum in the smaller volume up to a Michelin radius of 2.5 Å are also present in the new volume, up to a radius of 2.7 Å or larger. One would therefore expect that their contribution to the total spectrum remains the same, and that their contribution to the new affected spectrum only changes in intensity relative to the rest of the spectrum, but not in form. This is not in agreement with our findings and can only mean that the assumption that the solution spectrum (either for the whole box or for regions out to different *r*_*M*_-values as in Figure 6) is the weighted sum of just *two* spectra is too simple. In order to extract more detailed, local information, we now consider the solution spectra in a succession of sufficiently thin Michelin shells with increasing Michelin radii *r*_*M*_, i.e., the *local* spectra *D*_*solution*_^*loc*^(ω, *r*_*M*_).

**Figure 8:**
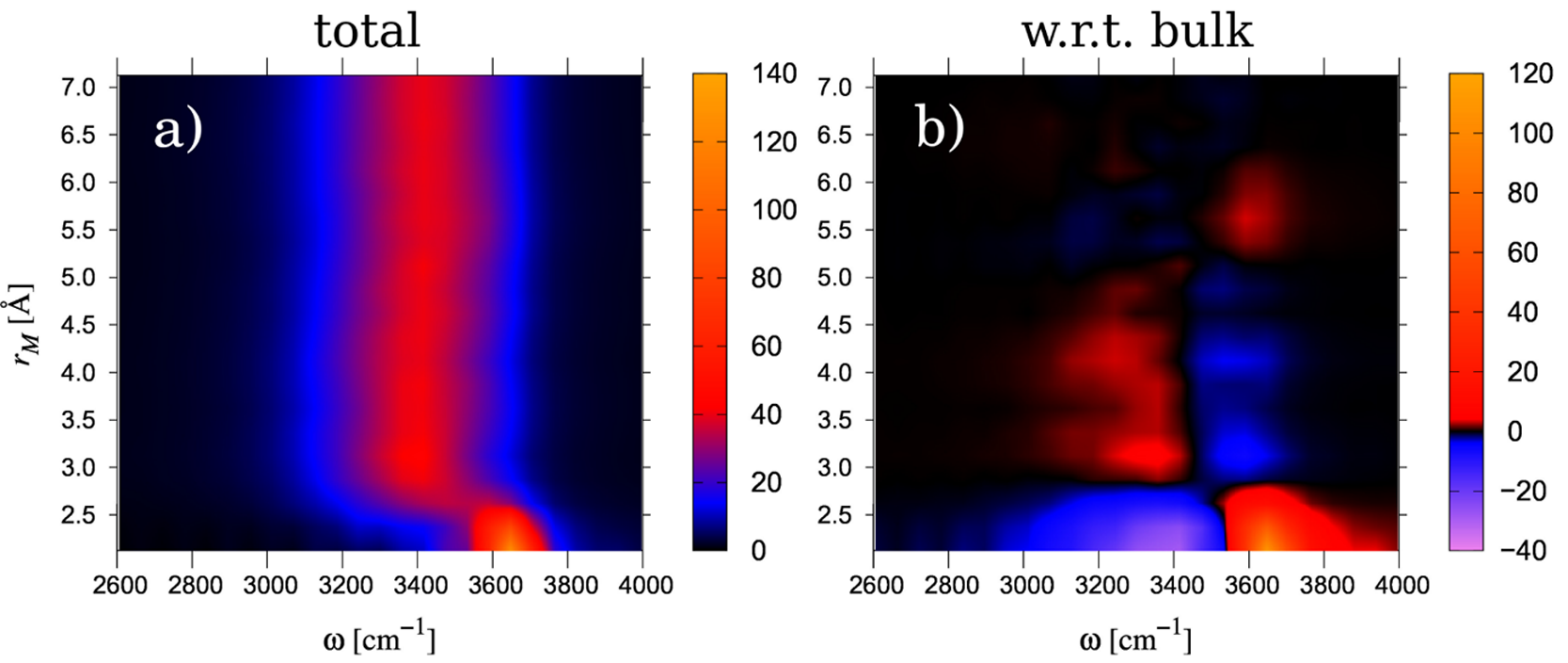
Spatially resolved spectra. (a) *D*_*solution*_^*loc*^(ω, *r*_*M*_) spectrum obtained by following oscillators for 375 fs, assigning the results to the bin where the hydrogen atom has spent most of its time. Bins are Michelin shells of thicknes *dr*_*M*_ = 0.25 Å. (b) Nonweighted difference spectra *D*_*solution*_^*loc*^(ω, *r*_*M*_) – *D*_*pure*_(ω).

To calculate power spectra for Michelin shells with thicknesses of 0.25 Å, we followed all individual hydrogen oscillators for a time span of 375 fs, calculated their spectrum in the usual way, and assigned each to the Michelin shell in which it resided most of its time. It is clear that if the time span to calculate the spectra is chosen too short, they will be sparsely sampled. On the other hand, if this time span is chosen too large, the oscillator will have visited several shells and have acquired spectra from each of them; in other words the calculated spectra will be smoothed along the radius *r*_*M*_. After some experimenting, we settled for the given values. The results are shown in Figure 8a, with *D*_*solution*_^*loc*^(ω, *r*_*M*_) being the spectrum attributed to the shell between Michelin radii *r*_*M*_ and *r*_*M*_ + 0.25 Å, and in Figure 8b, in the form of the difference *D*_*solution*_^*loc*^(ω, *r*_*M*_) – *D*_*pure*_(ω). Since the spectra are normalized to one oscillator, they do not depend on the thickness *dr*_*M*_ of the shell as long as this is small enough. The spectra are shown along the frequency axis (abscissa) in color code and are stacked along the Michelin radius (ordinate).

It is clearly seen that at distances below about 2.7 Å from the solute, the affected OH-oscillators on average oscillate at frequencies somewhat larger than where bulk water oscillates. In the difference spectrum this is seen as a positive peak at high frequencies and a negative peak at lower frequencies. These findings are the same as in Figure 6. We call the spectrum in this region an A-spectrum. Next, at distances between 2.7 and about 5 Å the opposite occurs; here the difference spectrum is negative in the high frequencies and positive in the low frequencies. We call the spectrum in this region a B-spectrum. This region corresponds roughly to the region where the tetrahedrality is high. It is interesting that the intensity of the B-spectrum is slightly decreased in shells near 3.7 Å, so we actually discern two B-spectra in a row. The distance where the separation occurs roughly corresponds to the end of the propulsion of hydrogen density near the waist in Figure 1. Although it is difficult to recognize the change from the first B-spectrum to the second in Figure 6, we do notice a dip in the plateau in Figure 5 at roughly this distance. Another surprising aspect is that beyond a distance of 5 Å the spectrum in Figure 8b inverts again, in the sense that now the high frequency intensity is again larger than in pure water; i.e., the spectrum becomes an A-spectrum again. Next, the difference spectrum gradually fades away, such that beyond about 6.5 Å there is hardly any difference anymore, in agreement with the results presented in Figure 5.

**Figure 9:**
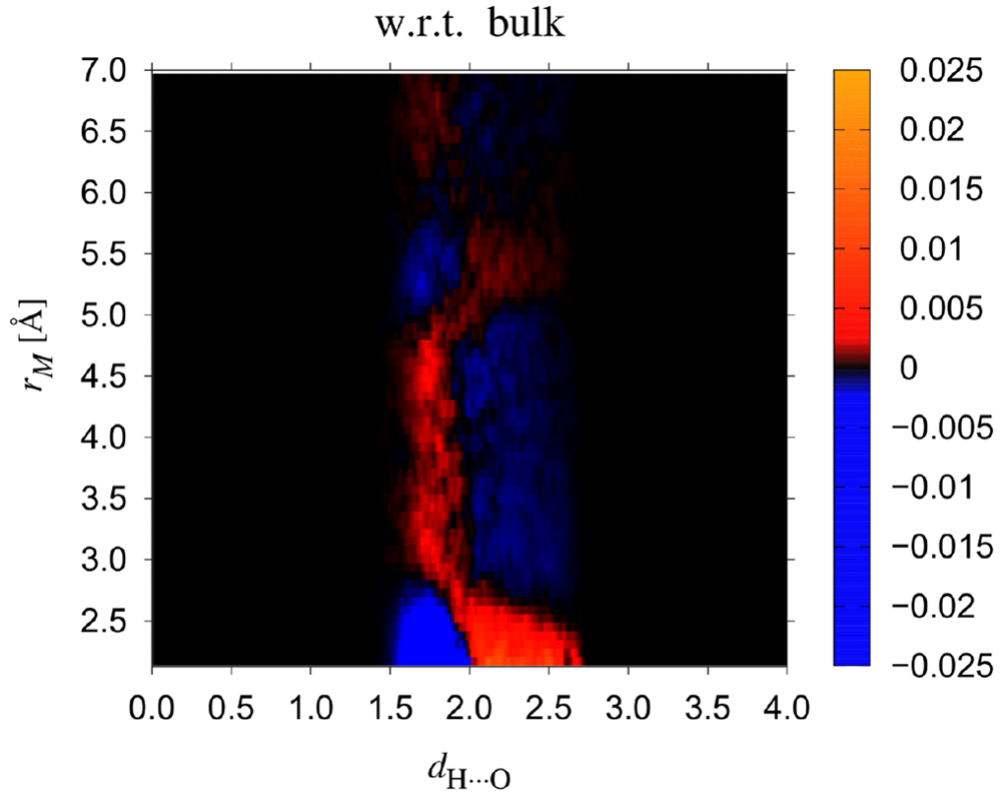
Probability density function to find hydrogen-bond lengths *d*_H···O_ for a given Michelin radius *r*_*M*_. The figure displays this probability, *P*(*d*_H···O_; *r*_*M*_), minus its bulk value *P*_*pure*_(*d*_H···O_). Results were averaged over Michelin shells of thickness 0.25 Å near the corresponding Michelin radii *r*_*M*_.

It is well-known that red-shifting (i.e., down-shifting) of the OH-vibrations in water occurs as a consequence of hydrogen-bonding, and usually the larger the shift is, the shorter the hydrogen bond is. As a measure of the length of the hydrogen bond, we take the distance *d*_H···O_ between the donated hydrogen atom and the accepting oxygen. This measure is slightly advantageous compared to using *d*_O···O_ as correlations involving the latter distance tend to be somewhat obscured by variations of the hydrogen bond angle. In Figure 9, we have plotted in color code the probability density function of the hydrogen bond length *d*_H···O_ minus the one for pure water, for various values of the Michelin radius *r*_*M*_ and stacked them along the vertical axis. For details see the figure caption. One clearly notices the correlation with the plots in Figure 8b, i.e. where the spectrum is downshifted, *P*(*d*_H···O_; *r*_*M*_) is shifted toward smaller values of *d*_H···O_. So we see the same ABBA structure as with the spectra. Using these data we have calculated the average values ⟨*d*_H···O_(*r*_*M*_)⟩, and found that in the two B parts of the hydration shell ⟨*d*_H···O_(*r*_*M*_)⟩ is smaller than its bulk value by about 0.03 Å, while in the thin A region nearest to the solute ⟨*d*_H···O_(*r*_*M*_)⟩ is larger than the bulk average by as much as about 0.5 Å. In the second A region, a very small elongation is discernible of about 0.01 Å.

We suggest that these results may be interpreted in the following way. In the first A spectrum, near the solute, the spectrum has excess intensity in the frequencies characteristic of a free OH-oscillator, indicating that hardly any hydrogen-bonding to the solute occurs. This agrees with our finding in Figure 7 that even those OH groups in this region, that according to our geometrical criterion qualify as being hydrogen-bonded to the solute, do not qualify as such by any reasonable vibrational spectroscopy criterion. We conclude that there is no appreciable hydrogen-bonding between the water molecules and the solute in this region. The second B spectrum in the region of *r*_*M*_ values between 3.7 and 5.2 Å coincides with a region where hydrogen densities are small and tetrahedrality is larger than average in pure water. Moreover, hydrogen-bonding appears to be stronger than in bulk water with foreshortened ⟨*d*_H···O_(*r*_*M*_)⟩ values compared to bulk water. On the basis of these findings one may stipulate that water in this region in some respects resembles water in ice. The second A region coincides with the second shell of excess hydrogen density and small tetrahedrality. We stipulate that these are signs that hydrogen-bonding is more difficult to achieve in this region and existing hydrogen bonds are somewhat weakened with respect to those in pure water. The first B region with *r*_*M*_ values between 2.7 and 3.7 Å is rather peculiar. Here the hydrogen density is large, while also tetrahedrality is large and the ⟨*d*_H···O_(*r*_*M*_)⟩ hydrogen bond distances are shortened. On the other hand, this Michelin region is less homogeneous in its properties than other regions. The water molecules near the solute's waist tend to donate hydrogen bonds to water molecules further out, and in the remaining part of the first B region, the water molecules to a large extent lie “flat”, i.e. along the Michelin surfaces.

## IV Summary and Conclusions

We have examined the perturbation that the almost-hydrophobic CO_2_ solute causes on the surrounding hydration shell, using AIMD simulations and a variety of analysis tools including power spectra for open systems. In particular, we presented space-resolved water OH vibrational spectra collected from gradually larger Michelin regions around the solute as well as from “slices” of the solution. Our analyses reveal a weakly oscillatory variation of not only the spectra but also of other properties, as a function of distance from the solute.

Thus, as for the static structural properties, we find that the distribution of water molecules around CO_2_, exemplified by the distribution of oxygen atoms, is influenced by the solute over length scales of a few times the range of repulsive forces and likewise for the hydrogen atoms as they are bound to live near the oxygen atoms. Properties like tetrahedrality and number of H-bonds per water molecule, but also the power spectra of the hydrogen oscillators, depend in principle on electrostatic and van der Waals interactions with the solute, and on local densities, i.e., indirectly on excluded volume effects.

We have given special attention to the water molecules very close to the solute, some of which give rise to a distinct high-frequency peak in the solute-affected spectra. Here we calculated uncoupled bond-stretching spectra to help decipher the power spectrum. Thus, assuming that all hydrogen atoms in this region move independently from each other, we interpret the power spectrum, in the range of frequencies corresponding to the internal dynamics of the water molecule, as being a superposition of smoothed delta peaks, each corresponding to an individual hydrogen atom at its particular position with respect to the solute. Using instantaneous frames from the AIMD trajectory as samples from the independent oscillator distribution we calculated the spectrum by stretching the O_w_H bonds in their full (and frozen) environments, and in each case, we calculated the *in situ* force constant of the OH bond. The OH oscillators were classified according to our geometric H-bond criteria as taking part in hydrogen bonds to water molecules, to the solute oxygen atoms, or just freely dangling; we found that the spectra of the last two populations hardly differ. This is an indication that the alleged hydrogen-bonding to the solute’s oxygen is based on accidentally meeting the geometrical hydrogen-bonding criteria, and not on corresponding changes in the electronic structure (frequency downshift), leaving only two OH classes close to the solute: the dangling ones and those that are truly H-bonded (to other water molecules).
